# Elevated Inter-Brain Coherence Between Subjects With Concordant Stances During Discussion of Social Issues

**DOI:** 10.3389/fnhum.2021.611886

**Published:** 2021-05-14

**Authors:** Christian Richard, Marija Stevanović Karić, Marissa McConnell, Jared Poole, Greg Rupp, Abigail Fink, Amir Meghdadi, Chris Berka

**Affiliations:** Advanced Brain Monitoring, Carlsbad, CA, United States

**Keywords:** hyperscanning, EEG, coherence, agreement, unanimity

## Abstract

Social media platforms offer convenient, instantaneous social sharing on a mass scale with tremendous impact on public perceptions, opinions, and behavior. There is a need to understand why information spreads including the human motivations, cognitive processes, and neural dynamics of large-scale sharing. This study introduces a novel approach for investigating the effect social media messaging and in-person discussion has on the inter-brain dynamics within small groups of participants. The psychophysiological impact of information campaigns and narrative messaging within a closed social media environment was assessed using 24-channel wireless EEG. Data were acquired from three- or four-person groups while subjects debated contemporary social issues framed by four scenarios of varying controversy: (a) investing in ethical vs. unethical corporations, (b) selecting travel destination based on social awareness, (c) determining verdict in a murder trial and the punishment of life in prison or death penalty, and (d) decision to vaccinate. Pre-/post-scenario questionnaires assess the effects of the social media information. Inter-brain coherence between subject pairs on each social issue discussed by subjects was analyzed by concordance, agreement vs. disagreement, and by group unanimity, unanimous vs. not unanimous. Subject pairs that agreed on the social issues raised in the scenarios had significantly greater inter-brain coherence in gamma frequency range than disagreeing pairs over cortical regions known to be involved in social interactions. These effects were magnified when comparing groups where subject pairs were unanimous in their stance on the social issues for some but not all scenarios. While there was considerable overlap between scenarios in what EEG channels were significant, there was enough variability to indicate the possibility of scenario-specific effects on inter-brain coherence.

## Introduction

Humans, and primates in general, are fundamentally social creatures. Our interactions through social media only happen to be the latest manifestations of our species’ gregariousness. Social media usage has grown exponentially within the last few years and has evolved into numerous interactive multimedia platforms such as Facebook, LinkedIn, Instagram, and YouTube. These platforms among others have created virtual communities connecting strangers and neighbors around the globe. Social media networks support the rapid formation of virtual peer groups where individuals can share information with others sharing common interests, however these virtual networks are often anonymous, and the experience of live social interaction can be lost. One approach to understanding the antecedents to the decision to share information and the impact of sharing on group behavior is to directly monitor the psychophysiological correlates of subject’s behavior on these social networks.

Social cognitive neuroscience and consumer neuroscience are beginning to explore the neurological mechanisms associated with the impact of information form and content on human decision-making (Milkman and Berger, 2014; Correa et al., 2015) using fMRI, fNIR, EEG, eye-tracking and other non-invasive brain monitoring technologies. These methods reveal the neural correlates of information-processing linked to decision-making and have been useful in predicting behavior (Lieberman, 2007; Tamir and Mitchell, 2012; Falk et al., 2013; Ramsøy et al., 2015; Scholz et al., 2017).

In contrast to single-person experimental designs that have dominated neuroscience (Astolfi et al., 2020), two-person or “multi-brain” neuroscience (Hasson et al., 2012) provides a framework to investigate the inter-brain dynamics that underlie social interactions (Liu and Pelowski, 2014). Montague et al. (2002) coined the term “hyperscanning,” the simultaneous quantification of inter-brain coupling across multiple participants, in their germinal paper inaugurating this new method for conducting research into the nascent field of social neuroscience. The term “Inter-Brain Coupling” is used here to refer to patterns of brain activity arising in two or more subjects as a consequence of their social interactions, and agnostic to the metrics used to quantify the degree of inter-brain coupling (e.g., phase locking value, coherence, phase synchrony index, etc.). The investigation of inter-brain coupling holds the promise of furthering our understanding of changes in brains associated with deficits or alterations in social cognition, such as of those with autism or schizophrenia, in therapeutic settings between therapist and client, in childhood development of elements underlying social cognition like empathy, and to improve teacher-student dynamics by providing otherwise unobservable information linked to social attention and engagement in the classroom.

Application of the hyperscanning methodology for investigations into social interaction have yielded insights into the neural underpinnings of shared goals, physiological characteristics of emergent leaders, and the degree of cooperation or collaboration in the group (Tognoli, 2008; Stevens and Galloway, 2016; Balconi et al., 2017; Berka and Stikic, 2017; Czeszumski et al., 2020; Wang et al., 2020). Physiological synchrony in electroencephalograms (EEG) and electrocardiograms (ECG) between members of the same group have been shown to be predictive as to whether members are competing or cooperating as well as with consensus or success in team performance. This approach has been applied to a wide range of dyad and small group interactions including computer/VR gameplay, musical performance and other visual-motor exercises, dialogs between couples or student teams, and real-world applications including flight and submarine team simulations (Astolfi et al., 2011, 2012; Kawasaki et al., 2013; Toppi et al., 2016; Balconi et al., 2017; Berka and Stikic, 2017; Stevens et al., 2018; Czeszumski et al., 2020).

Cooperation in a Prisoners Dilemma game was associated with increased frontal theta and alpha coherence while competition showed decreased synchronization across the frequency bands (Astolfi et al., 2009, 2010, 2011). Musicians playing together show increased synchronization in delta and theta (Sänger et al., 2012). In a task where subjects take turns imitating nonsense hand gestures, greater synchronization is observed across multiple frequencies (Dumas et al., 2010; Kawasaki et al., 2013). In a flight simulation, researchers found that frontal and parietal regions of pilot and co-pilot brains exhibited the greatest hyperconnectivity during the most challenging tasks, take-off and landing (Toppi et al., 2016).

Kinreich et al. (2017) compared inter-brain coupling of romantic couples with pairs of strangers engaged in a naturalistic setting, planning a “fun day” with each other. Inter-brain coupling was higher in romantic couples compared to strangers, particularly manifest in gamma activity (38–42 Hz) at frontal and temporo-parietal regions. Increased inter-brain coupling at gamma was anchored in nonverbal social behavior, increasing during moments of social gaze and marginally higher when individuals expressed positive affect. These effects were seen in strangers as well as romantic couples. The author concluded that gamma activity appears to be an important correlate of emotional attachment and the degree of social connectedness between partners.

The gamma frequency band has been implicated of differential effects of cooperation vs. competition during a reaction time task. Inter-brain synchrony was selectively increased between pairs that were playing cooperatively, but not during the competitive trials (Barraza et al., 2020). These transient increases in brain-to-brain gamma activity during cooperation appeared just prior to and immediately following presentation of the stimulus. The cooperation-associated increases in inter-brain gamma activity were clustered between left frontal-temporal EEG channels.

The goals of this research project were to investigate the psychophysiological impact of various social media messaging on both individual behavior and decision-making (single-person neuroscience) and inter-brain dynamics between subjects during discussion of social issues taken from a set of real-world scenarios (two-person neuroscience). This paper focuses on the latter component of the project. Here we report our findings specifically on the neurodynamics of small group discussion vis-à-vis the concordance between subjects over the stances they took on social issues framed by four different social scenarios. We hypothesized that agreeing pairs would have greater inter-brain coherence than disagreeing pairs in gamma frequency range at cortical regions that have been previously associated with social interactions.

## Materials and Methods

### Participants

159 healthy participants between the ages of 18–40 years old were enrolled in the study. Participants were recruited through online postings (Craigslist, Indeed, and Facebook) around San Diego County. Interested participants were screened through an initial telephone questionnaire to determine eligibility. Participants were excluded if they reported a diagnosis for any of the following: sleep, psychiatric, neurological, eating, behavioral (including Attention Deficit Disorder), or cardio-pulmonary/vascular disorders; uncontrolled blood pressure; heart disease; and/or HIV+/AIDS. Additional exclusion criteria included head trauma within the past 5 years, regular use of prescription drugs that can alter EEG or impair their ability to participate, use of illegal drugs (recreational and medical marijuana users were not excluded), excessive use of nicotine, alcohol and/or caffeine, untreated vision or hearing issues, pregnant or nursing, and inadequate familiarity with the English language. Protocols were approved by Alpha IRB and Air Force Research Laboratory (AFRL).

### Equipment

Each computer workstation was fitted with Social Media Analytical Replication Toolkit (SMART) social media simulation software (Cubic Corp, Austin, TX, United States), and Pro Nano Eye-Tracker eye-tracking and pupillometry camera and software (Tobii, Stockholm, Sweden). The SMART platform developed by the Intific Division of Cubic Defense Applications (CDAI) allows users to interact within a closed social media environment for experimentation and real-time exercises. This specific design of the SMART platform was to mimic the popular social media sites, Twitter, and Facebook.

For the SMART platform, we gathered two types of content for participants to browse, noise and curated content. Noise content was meant to make the platform more realistic, and included photography content from well-known companies, actors, musicians, television personalities, and animals were handpicked from various factual Twitter feeds and added to the SMART platform. Unlike noise content, the curated content was related to one of four real-world scenarios social issues of interest. Curated content included real video and news articles that were hand selected from a variety of mainstream news outlets. Each scenario featured Curated Content from both viewpoints (positive and negative tone toward topic) to ensure there was no bias toward either side.

### Scenarios

Four scenarios based on real-life issues were created: (1) investing in perceived ethical vs. unethical corporations, (2) selecting travel destination based on social awareness and commitment to assisting people in need, (3) death penalty decision to access deeply held beliefs or “sacred” values and, (4) contemporary health issue (decision to vaccinate). All participants in a group would be exposed to the same scenario at the same time but interacted with them individually on the SMART platform. Participants were instructed to scroll through each scenario-specific “social media” site and to interact as they would if this was their personal social media account. Prior to starting each scenario, participants were administered a scenario specific questionnaire. These questionnaires were used to quantitatively gauge how much social media impacts their decision-making as well as the effects of a group discussion with his or her peers. They provide a brief description of the scenario followed by a set of questions. These questionnaires are also administered after the participant finishes the specific scenario and after the final group discussion.

#### Scenario One–Company Ethics

In scenario one, participants are given a specific amount of money that they can invest into a variety of stocks. They may choose from a list of 6 companies. Three of these companies (Hilton, Volvo, and T-Mobile) have been rated as ethical companies, and the other three companies (Chick-Fil-A, Uber, and United Airlines) have had many public unethical issues within recent years^1^. The goal of the scenario is to see which companies (ethical or unethical) the participant wishes to invest in after being exposed to the various types of Curated Content.

#### Scenario Two–Free Travel Destination

In Scenario two, participants are told that they are getting a hypothetical all-expenses paid 1-week vacation and had to choose between either Paris, France or Sulawesi Island, Indonesia. There were both potential risks and rewards associated with travel to these locations. Paris had been contending with large protests by the Yellow Vest Movement and sporadic violence. Sulawesi Island had recently been hit by a destructive tsunami and while safe to travel to, was still rebuilding. Selection of Sulawesi Island offered an extra opportunity for the participant to volunteer to help rebuild what has been destroyed. If they chose to volunteer, the participant would have to designate how many hours and days of their vacation they wish to aid relief efforts.

#### Scenario Three–Murder Trial and Punishment

In Scenario three, participants are informed that they are part of the jury for the trial of a young man, who is accused of murder (victim: a female University of South Carolina college student), based on a true story in the media. Participants received information from both sides and were asked to decide on a verdict. If they found the defendant guilty, they were asked for their stance on death penalty vs. a long prison term. The participants are also given information from both sides of the current debate of capital punishment in the state of California, which had recently placed a moratorium on death penalty sentencing.

#### Scenario Four–Vaccinations

In the final scenario, participants are asked to imagine they are the parent to two young children; the first was vaccinated and while they are healthy, they have been showing some developmental delays (such as verbal and motor skills). Their doctor has informed them it is now time to vaccinate their second child with the same vaccines and they must decide whether to vaccinate their second child or not. Participants are presented with various types of articles and videos that present information that is for and against vaccinations.

### Procedures

Eligible participants were scheduled in groups of three or four and were asked to arrive at the Advanced Brain Monitoring (ABM) office in Carlsbad, CA for either an 8 a.m. or 9:30 a.m. starting time depending on the study session. Participants were instructed to get a good night’s sleep the night prior to their study visit, and to abstain from caffeine during their testing day. Participants completed paperwork to document their consent to be part of this study; consenting participants were then administered a battery of personality and behavioral questionnaires (NEO, POMS, etc.) during which they were fitted with a B-Alert^TM^ X24 EEG sensor headset.

Participants in each group progressed through three stages: (1) acquisition of EEG during a standard set of neurocognitive tasks, (2) interaction with curated social media content via the SMART platform, and (3) a discussion period during which group participants interact in conversational reviews of each scenario issue. The first stage began upon completion of intake questionnaires with all participants engaging in a series of neurocognitive tasks to measure active and passive vigilance, sustained attention, processing speed, emotional responses, and memory (Berka et al., 2004, 2005, 2007; Stikic et al., 2014; Waninger et al., 2018). Once participants completed all neurocognitive tasks, they were set up to interact with the SMART platform. Social media accounts were created for each of the group participants which allowed the participant to like, share, or retweet posts as they normally would do on their personal social media accounts. Participant interactions with SMART were not broadcasted to other group participants’ accounts. Group discussion commenced once all group participants finished navigating through the SMART platform content. Group participants did not meet until the start of discussion. The discussion lasted a minimum of 20 min, allowing at least 5 min of conversation for each scenario. Each scenario was addressed in the same order for all groups during the discussion period. A research technician served as a moderator to facilitate discussion of the scenario issues. questions. Participants were asked the same questions for each scenario: “What did you decide and why?” and “What, if any, social media posts influenced your decision?” During the discussion, another technician time-stamped EEG with event markers to indicate the start and end times for each scenario discussion. When the discussion had ended, the participants were instructed to complete scenario-specific questionnaires one last time before completing their participation in the study.

### Behavioral and Physiological Measures

We used subject responses on scenario-specific questionnaires to determine the social concordance of subject pairs, i.e., whether pairs agreed or disagreed on a given scenario issue, and to determine whether there was unanimity in the stances held by members of the same discussion group. EEG and ECG were concurrently acquired using the B-Alert^®^ X24 wireless sensor headset (Advanced Brain Monitoring, Inc., Carlsbad, CA, United States). This system has 20-channels of EEG located according to the International 10–20 montage at Fz, Fp1, Fp2, F3, F4, F7, F8, Cz, C3, C4, T3, T4, T5, T6, O1, O2, Pz, POz, P3, and P4 with an auxiliary channel for ECG. ECG electrodes were placed on the right and left clavicle. Linked reference electrodes were located behind each ear on the mastoid bone. Recordings were sampled at 256 Hz with a high band pass at 0.1 Hz and a low band bass, fifth order filter, at 100 Hz obtained digitally with Sigma-Delta A/D converters. Amplification and the A/D conversion was done adjacent to the electrode sites, allowing for high quality data to be collected with impedance cut-offs at or below 40 kΩ. Each computer workstation was fitted with eye-tracking and pupillometry camera and software (Tobii Pro Nano Eye-Tracker, Stockholm, Sweden). Eye-tracking equipment was calibrated to ensure that accurate pupillometry data would be captured.

### Data Analysis

Data were transmitted wirelessly via Bluetooth to a host computer, where acquisition software then stored the physiological data. All preprocessing operations on these data were performed in MATLAB using EEGLAB tools. Data were bandpass filtered (1–40 Hz) before conducting artifact removal. Independent component analysis (ICA) was performed on all channel data for each subject. The ICLabel function was used to reject components classified as having sources other than brain, such as from line or channel noise, eye blinks, lateral or vertical eye movements, and other myological sources. ICLabel uses a classifier that is pre-trained by thousands of labeled components obtained through crowdsourcing (Pion-Tonachini et al., 2019). Power spectral densities (PSD) were then computed for each 1 s epoch by averaging Fast Fourier Transforms of the EEG segment corresponding to each epoch and to the two EEG segments immediately preceding and following that share 50% overlap with the given epoch. Kaiser window was applied to the EEG segments prior to Fast Fourier Transform. Frequency bands were defined as Delta (1–3 Hz), Theta (3–7 Hz), Alpha (8–13 Hz), Beta (13–30 Hz), Fast Beta (21–30), and Gamma (25–40 Hz).

Inter-brain coherence (IBC) was calculated between all possible subject pairs within each discussion group using the coherence equation:

$$I\,B\,C_{x\,y}\,\left(f\right)=\frac{{\left|D_{x\,y}\,\left(f\right)\right|}^{2}}{D_{x\,x}\,\left(f\right)\,D_{y\,y}\,\left(f\right)}$$

on the same EEG channel in both subjects, where D_*xy*_(f) is the cross power spectral density function between subject pairs, *x* and *y*. Event-markers set at the transitions between scenarios during group discussion were used to extract scenario-specific subsets of EEG recordings. Scenario-specific IBC was calculated between subject pairs from the same discussion group, i.e., those that had socially interacted with each other.

IBC was analyzed at each EEG channel by social concordance of subject pairs (agree vs. disagree), and by group unanimity (shared stance vs. mix of stances) at six frequency bands (delta 1–3 Hz, slow theta 3–5 Hz, fast theta 5–8 Hz, slow alpha 8–10 Hz, fast alpha 10–12 Hz, beta 12–30 Hz, and gamma 25–40 Hz). Subject pairs were excluded if the scenario stance was missing for one or both participants.

Sample sizes were not expected to be equal given that subject membership in either agreeing or disagreeing groups was self-selecting. Welch’s *t*-test was selected to mitigate unequal sample sizes; test was applied to each EEG channel for the statistical analysis of pair agreement and group unanimity. We report EEG channels significant at or below α, and after FDR correction (Benjamini and Hochberg, 1995). Significance was set at *α* = 0.05.

## Results

### Behavioral Measures

**FIGURE 1:**
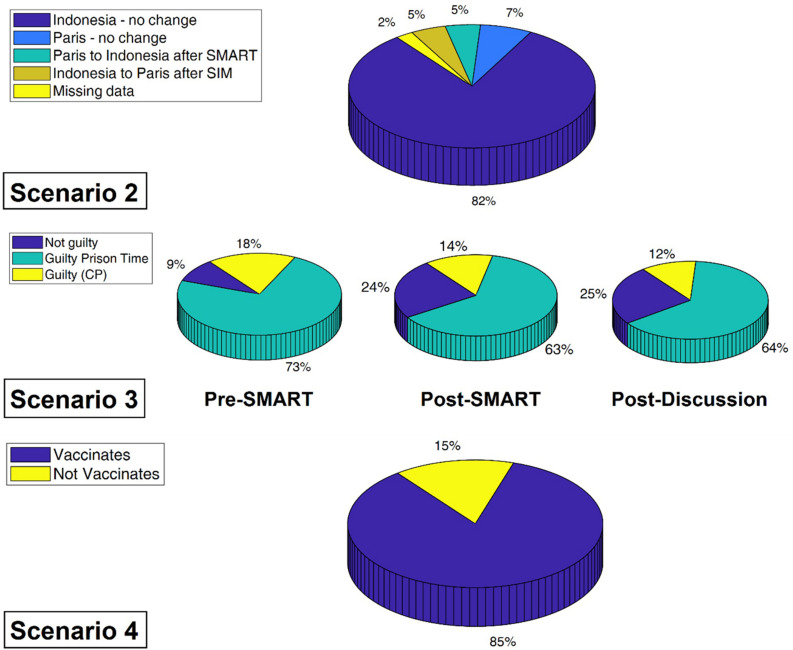
Percent of participants holding each stance on scenario issues.

The percentage of subjects that changed their stance on issuesbetween the three time periods varied by scenario (Figure 1 and Table 1). In scenario 2, participants’ decision on volunteering significantly increased after interacting with the SMART platform (Post-SMART). There was a significant increase in volunteering hours post-SMART [3.4 h, p(127) = 0.0001], and a non-significant increase after discussion (0.5 h). Subjects were most equivocal on the verdict issue of scenario 3 with large numbers changing their stance after interacting with the content on the SMART platform and following the group discussion. The percentage of those who held the “Not Guilty” stance increased from 9 to 24% after SMART. 71% answered “Guilty” and did not change their answers throughout the experiment. In scenario 4, only five (5) participants had any change in their opinion on vaccination. Following interaction with the SMART platform, 3% of participants changed from pro-vaccination to anti-vaccination, and <1% changed from anti- to pro-vaccination. After the discussion period was over, the only shift in participants’ stances were from anti- to pro-vaccination.

**TABLE 1:** Sample sizes of subjects’ stances by scenario at pre-SMART, post-SMART, and post-discussion periods.

	**Pre-SMART**		**Post-SMART**		**Post-discussion**	
	**Agree**	**Disagree**	**Agree**	**Disagree**	**Agree**	**Disagree**
Scenario 2–travel destination	75	31	76	30	77	29
Scenario 3–death penalty	59	29	43	21	52	17
Scenario 3–verdict	90	16	67	39	81	25
Scenario 4–vaccinations	78	29	73	34	75	32

### Inter-Brain Coherence by Social Concordance

**FIGURE 2:**
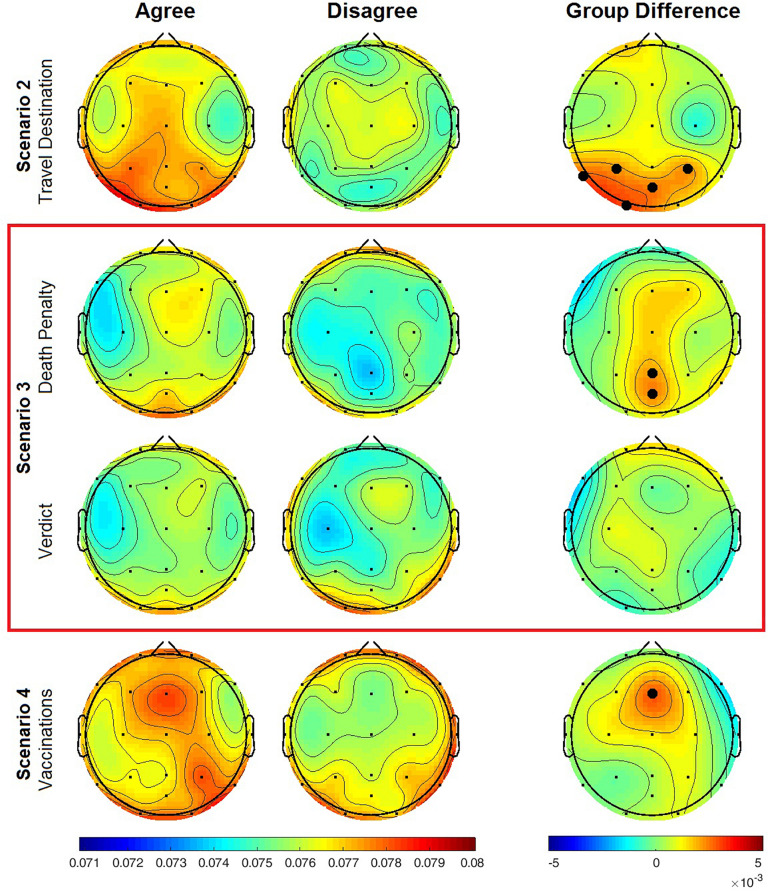
Inter-brain coherence by social concordance in gamma frequency band during each scenario. IBC topological maps for agreeing and disagreeing pairs depicted in columns on left with difference between groups in rightmost column. Color bar on bottom left is for agree and disagree groups; bottom right side color bar applies to group difference topological maps with range [-0.0005 to +0.0005]. Red box contains maps for the two issues in scenario 3, concordant or discordant stances on death penalty, and the guilty or innocent verdict for alleged murder suspect. Sample sizes are for number of subject pairs; scenario 2, agree, *N* = 77, disagree, *N* = 29; scenario 3 death penalty, agree, *N* = 52, disagree, *N* = 17; scenario 3 verdict, agree, *N* = 81, disagree, *N* = 25; scenario 4 vaccination, agree, *N* = 75, disagree, *N* = 32. EEG channels significant at α denoted by black filled circle. Gamma frequency range, 25–40 Hz.

In our measures of social concordance between subject pairs, we found greater average IBC in agreeing over disagreeing pairs in the gamma frequency band (25–40 Hz). The largest significant differences between the two groups were distributed across temporo-parietal regions, but also found at left occipital and frontal midline channels (Figure 2). Significant differences between the two social concordance factor levels (agree vs. disagree) during discussion of the travel destination issue of scenario 2 were concentrated in temporal, parietal, and occipital EEG channels (Table 2). Discussion of the two issues in scenario 3, the death penalty and murder case verdict, was associated with similar spatial patterns of IBC by social concordance, but IBC was significantly higher in agreeing over disagreeing subject pairs only for the death penalty issue. During discussion of vaccinations (scenario 4), agreeing subject pairs exhibited significantly greater IBC over frontal midline (Fz). Average IBC among agreeing pairs was elevated in the right temporo-parietal region situated under channels P4 and T6 although not significantly so compared to disagreeing pairs. Inter-brain coherence appears to have scenario-specific variation in spatial patterns of IBC for both agreeing and disagreeing subject pairs with the two issues for scenario 3 looking more like each other than IBC during discussion of scenarios 2 and 4.

**TABLE 2:** Significant IBC in socially concordant subject pairs.

	**Channel**	**t-statistic**	**d.f.**	***p*-value**
Scenario 2–travel destination	T5	2.3376	44.7361	0.0239
	P3	2.1038	44.0962	0.0411
	P4	2.1038	66.8695	0.0407
	POz	2.3893	52.6218	0.0205
	O1	2.7279	54.4895	0.0086
Scenario 3–death penalty	Pz	2.0551	34.1881	0.0476
	POz	2.3282	24.9933	0.0283
Scenario 4–vaccinations	Fz	2.9013	81.6523	0.0048

### Inter-Brain Coherence by Group Unanimity

**FIGURE 3:**
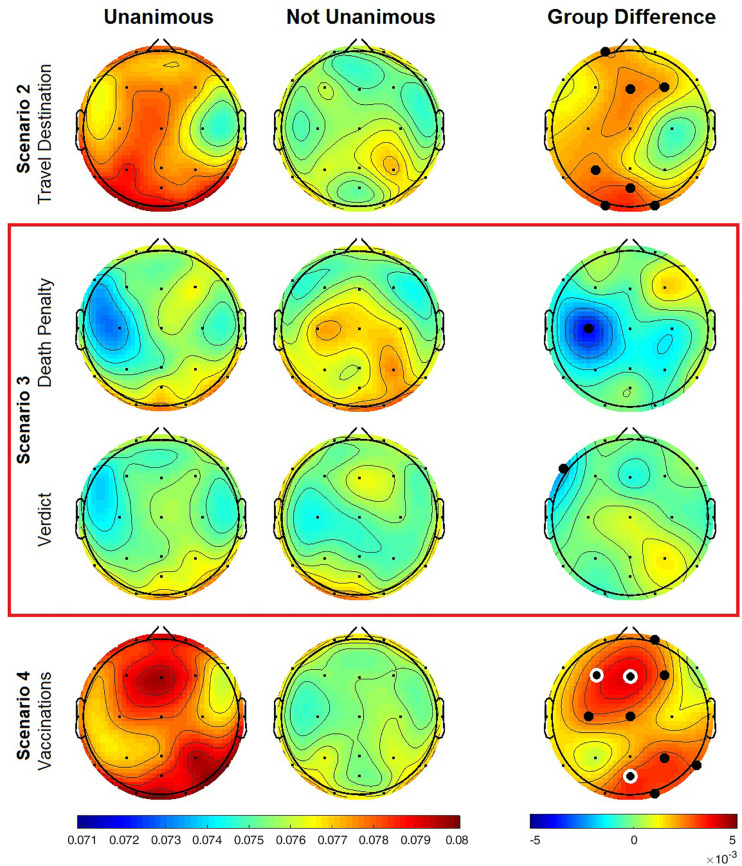
Inter-brain coherence in gamma frequency band during each scenario by group unanimity. Red box contains topological maps for the two issues in scenario 3, concordant or discordant stances on death penalty, and the guilty or innocent verdict for alleged murder suspect. Sample sizes are for number of subject pairs. Significant EEG channels at *α* = 0.05 denoted by black filled circle, those marked with white ring are significant after FDR correction for multiple comparisons.

Analysis of IBC by unanimity was conducted to account for the effect of all subjects in a group on each other to complement the more granular pairwise concordance in stances (agree vs. disagree). Spatial patterns of IBC were like those found in the social concordance analysis with greater IBC in unanimous vs. non-unanimous groups (Figure 3). Groups that were unanimous on the scenario 2 issue were found to exhibit significantly elevated IBC, concentrated across left fronto-parietal and occipital regions (Table 3). A similar pattern for group unanimity was found in scenario 4 with significant differences appearing in midline frontal and right temporo-parietal regions. Groups that were unanimous in the scenario 3 issues had significantly lower IBC in the left fronto-central region compared to non-unanimous groups. The number of unanimous and non-unanimous groups, each comprised of 3 or 4 subjects, are reported in Table 4. The total number of groups was 51, but only 37 groups had the minimum of 3 subjects required for group analysis.

**TABLE 3:** Significant IBC in group unanimity.

	**Channel**	**t-statistic**	**d.f.**	***p*-value**
Scenario 2–travel destination	Fp1	2.1064	84.6982	0.0381
	Fz	2.4129	77.1746	0.0182
	F4	2.2276	84.9538	0.0286
	P3	2.4461	84.9902	0.0165
	POz	2.7765	84.1826	0.0068
	O1	2.6743	84.0551	0.009
	O2	2.6338	84.0232	0.01
Scenario 3–verdict	F7	-2.291	81.4025	0.0245
Scenario 3–death penalty	C3	-2.6275	24.6365	0.0146
Scenario 4	Fp2	1.9936	84.4153	0.0494
	F3	2.9839	71.3402	0.0039
	Fz	3.6058	70.128	0.0006
	F4	2.3251	70.423	0.023
	C3	2.2206	81.4672	0.0292
	Cz	2.1192	79.2102	0.0372
	T6	2.2255	66.3784	0.0294
	P4	2.5031	60.5305	0.015
	POz	2.929	67.2731	0.0046
	O2	2.369	75.7466	0.0204

**TABLE 4:** Sample sizes of unanimous and non-unanimous groups by scenario.

	**Always agreed**	**Always disagreed**	**Agreed pre-SMART**	**Agreed post-SMART**	**Agreed post-discussion**
Scenario 2	16	16	18	19	20
Scenario 3	16	6	31	16	27
Scenario 4	17	19	18	18	19

## Discussion

Our investigation into the effects of social concordance on inter-brain coherence has provided evidence in support of two primary conclusions: (1) that our results are largely in support of inter-brain coupling at the gamma band as a biomarker for social concordance manifest here when subject pairs agreed on scenario issues, and (2) that patterns of IBC appear to be sensitive to informational context and content based on IBC variability between the different scenario issues.

This study replicated results from previous studies implicating inter-brain gamma activity with core elements of social cognition that underlie social connectedness (Kinreich et al., 2017; Barraza et al., 2020). In our measures of social concordance, we found greater average IBC in agreeing over disagreeing subject pairs (Figure 2). Significant differences between the two groups were distributed across temporo-parietal regions including left occipital channel, and frontal midline. The spatial distributions of inter-brain coherence divided by group unanimity were like those found in the analysis by social concordance (agreeing vs. disagreeing subject pairs), albeit with more robust differences between unanimous and non-unanimous discussion groups (Figure 3).

Our results are largely consistent with other reports on the relationship between inter-brain gamma activity and social connectedness. A recent study found that inter-brain synchrony in the gamma band was selectively increased between subjects that were playing cooperatively during a reaction time task but not during the competitive trials (Barraza et al., 2020). They found that transient increases in brain-to-brain gamma activity during cooperation appeared just prior to and immediately following presentation of stimulus. These cooperation-associated increases in inter-brain gamma activity were clustered between left frontal-temporal EEG channels, leading the authors to conclude that this pattern of gamma activity in temporo-parietal networks is a biomarker of shared intentionality between subjects. Positive interactions in mother-child dyads were associated with inter-brain coupling between their superior temporal sulci (STS) also in the gamma band (Levy et al., 2017), a region implicated in speech perception, prosody, and phonological processing. Another study comparing inter-brain coupling between romantic couples and strangers engaged in a naturalistic setting, planning a hypothetical “fun day” with each other, found greater inter-brain coupling for romantic pairs in the gamma range (38–42 Hz) across frontal and temporo-parietal regions (Kinreich et al., 2017). The increased inter-brain coupling at gamma was also found to be linked to the duration of social gaze and instances of shared positive affect between strangers as well as romantic pairs.

Research on this topic is ongoing, but the handful of studies specifically investigating the inter-brain activity underlying measures of social connectedness are not completely in agreement. Another recent hyperscanning study, of debate between Tibetan Buddhist monks, van Vugt et al. (2020) reported equivocal evidence for increased inter-brain synchrony when monk dyads were in agreement on claims raised during theological debate. This increase was found in frontal EEG channels within the alpha frequency band (10–14 Hz), and the effect was reversed in their replicate study showing disagreement rather than agreement on debate points was associated with an increase in frontal alpha inter-brain synchrony. While the reason for the discrepancy was not explicitly addressed, they do mention that unlike the initial exploratory study, the debate in the replicate study was restricted to only one topic raising the possibility that the topic on which subjects agreed or disagreed could influence the strength of shared inter-brain gamma activity.

Only the verdict issue of scenario 3 showed no significant changes to gamma band IBC between agreeing subject pairs (Figure 2). If it is the case that greater inter-brain synchrony at higher frequencies is a reliable biomarker of social concordance, then its absence in the verdict issue might reflect how subjects differentially weighed their concerns over the details of the case, the level of uncertainty subjects contended with in determining the verdict they arrived at, or possibly even reflecting the confidence in their decision. Uncertainty has been shown to activate frontal and parietal cortices (Huettel et al., 2005).

It is also possible that variability in the emotional impact of a topic can shape inter-brain activity. For example, another study found that emotional impact of “hyper-unfair” behavior between human subjects was correlated with inter-brain density (IBD), the number of significant inter-brain connections, with emotional impact increasing in proportion to IBD (Ciaramidaro et al., 2018). The analyses conducted for our report were limited to inter-brain coherence between subjects at the same EEG channels, e.g., comparing subject 1 Cz with subject 2 Cz. While we did not calculate IBD in our study, the scenario issues vary in how controversial they are, and as such, vary in how effective they are at evoking strong emotional responses. Viewed this way, the variability in IBC by scenario issue supports the possibility that IBC is sensitive to informational context, and is consistent with earlier reports providing evidence that the structure of social interaction itself can modulate inter-brain effects (Liu and Pelowski, 2014). We might expect reduced or non-existent IBC when pairs of subjects disagree. It remains unanswered to what degree the scenario-specific content contributes to the differences in inter-brain coherence between scenarios (Liu and Pelowski, 2014).

In summary, we found greater inter-brain gamma activity between subjects in agreement during discussion of a set of social issues, and in discussion groups where subjects were unanimous on a given issue. These effects were most prominent in temporo-parietal and frontal cortical regions for both the concordance and unanimity analyses, regions that are known to mediate social interactions. These results lend further support to the hypothesis that shared intentionality is reflected in inter-brain coupling within gamma frequency band.

## Data Availability Statement

The raw data supporting the conclusions of this article will be made available by the authors, without undue reservation.

## Ethics Statement

The studies involving human participants were reviewed and approved by Alpha IRB. The patients/participants provided their written informed consent to participate in this study.

## Author Contributions

CB, MM, and GR designed experiment. MM, GR, JP, and AF gathered the data. CR analyzed the data. CR and CB wrote the manuscript. All authors contributed to the article and approved the submitted version.

## Conflict of Interest

All authors are employed by Advanced Brain Monitoring. CB is also a shareholder in Advanced Brain Monitoring.

## Footnotes

1 https://www.worldsmostethicalcompanies.com/honorees/
